# New Prospects for Research on Manipulation of Insect Vectors by Pathogens

**DOI:** 10.1371/journal.ppat.0020072

**Published:** 2006-07-28

**Authors:** Thierry Lefèvre, Jacob C Koella, François Renaud, Hilary Hurd, David G Biron, Frédéric Thomas

**Affiliations:** Scripps Research Institute, United States

A growing number of studies demonstrate, or suggest, that vector-borne parasites manipulate phenotypic traits of their vectors and hosts in ways that increase contacts between them, and hence favour the parasites' transmission [1,2]. Understanding these processes is not only exciting for purely scientific reasons but also important because of their role in applied parasitology, such as epidemiology and medicine. The most frequently reported changes induced by vector-borne parasites are alterations of biting rates in vectors or of attractiveness in vertebrate hosts [3,4]. Our aim here is to elaborate further on some potentially interesting and important avenues for future research in this area. We begin this paper with a brief overview of the main mechanisms used by vectors to locate their vertebrate host, as it helps to grasp the fundamentals of the research on manipulation in vectors, as well as its current challenges.

Bloodsucking insects have well-developed sensorial machinery to locate and choose their host [5]. Host location behaviour is usually organized into three areas which show considerable variation among vector species: (i) the appetitive search, (ii) the activation and orientation, and (iii) the attraction. The two last steps involve insect responses to external stimuli, mainly visual and odour cues, but also heat and to a lesser extent, water vapour and sound [5]. Vision is most widely used by diurnal insect vectors (e.g., blackflies, tsetse flies, several mosquitoes). The detection depends mainly on differences in colour contrast and intensity contrast; generally, flies are attracted to blue/black objects while they are repelled by yellow ones. Odour-mediated host-seeking has been more thoroughly studied and seems to be utilized by virtually all bloodsucking insects. The olfactory stimuli used by the insects are various, ranging from carbon dioxide to lactic acid, ammonia, acetone, octenol, phenolic components of urine, and sweat. Bloodsucking insects can be also very sensitive to heat [5]. Although some of these components (vision, olfaction, hearing) could be theoretically altered by parasites in ways that may be predicted to enhance parasite transmission, only a few have been considered.

## 

### Bite more or bite better?

Qualitative manipulation, according to which generalist bloodfeeding insects, once infected, would develop a feeding preference for hosts targeted by the parasite, is an underexplored scenario. Maximising transmission towards a suitable host could be achieved by parasites by inducing in the vector a sensory bias for host traits that are correlated with optimal suitability for the parasite. Qualitative manipulation could theoretically occur at two levels: (i) at the interspecific level, with infected vectors biting more than expected on suitable host species for the parasite and (ii) at the intraspecific level, when infected vectors prefer feeding on less-immune hosts or on individuals that are uninfected (and thus do not yet harbour potential competitors). In particular cases, however (e.g., *Plasmodium*), the reverse tendency might be expected in order to find a sexual partner of a different strain. To test for the qualitative manipulation hypothesis, a dual-port olfactometer could be used to quantify the behavioural responses of infected and uninfected insect vectors to volatiles emitted by different host species. For instance, *Glossina palpalis gambiensis* has a broad range of hosts in central Africa (humans, reptiles, bushbuck, and ox) and is the main vector of *Trypanosoma brucei gambiense* responsible for the medically important Human African trypanosomiasis. We would predict that once infected, flies are more attracted by human cues than by those of other vertebrates.

**Figure:**
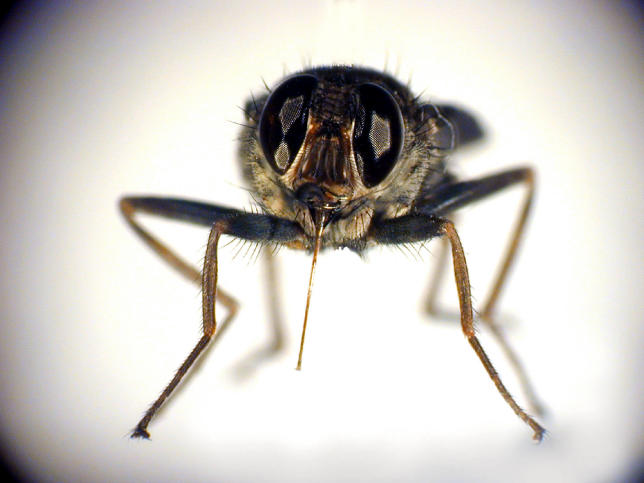
Glossina morsitans morsitans is the vector of several Trypanosoma species responsible for human African trypanosomiasis and animal African trypanosomiasis (nagana). (Photograph submitted by Michel Dukhan)

### Betrayed by smell.

All kinds of diseases are associated with changes in the infected individual's odour profile [6–9]. These changes have generally been considered as nonspecific symptoms of infection with no adaptive value. However, since the publication of several recent studies [10–13], and especially Lacroix et al. [2] who showed that people infected with transmissible stages of malaria produced something attractive to mosquitoes, it seems clear that more research should be performed to explore the hypothesis that alterations in odour profiles could be adaptive manipulative changes exerted by vector-borne parasites to increase their transmission. Along the same idea, the hypothesis according to which parasites inhibit some of the processes through which certain hosts are less detected/chosen than others [14] deserves consideration. Experimental tests of these hypotheses could be based on the same device as before (i.e., a dual-port olfactometer) combined with a gas chromatography–electroantennograph approach to detect and identify allomone/kairomone eventually emitted by infected and uninfected vertebrates.

### Going beyond behaviour.

In addition to greater consideration of the proximate mechanisms mediating parasite-induced changes in feeding behaviour, further research might benefit from also considering traits other than behavioural ones. Reduction of fecundity has been reported frequently in *Plasmodium*-infected mosquitoes [1]. Altering vector resource management may increase available nutrient reserves or avoid the cost of laying eggs, which in turn could enhance the vector's longevity and hence the parasite's overall transmission. Further experiments are clearly needed to confirm this interesting hypothesis [1]. Protozoan pathogens such as *Plasmodium, Leishmania,* and *Trypanosoma* also are able to evade the immune system of their vertebrate hosts by, for example, penetrating and multiplying within cells, varying their surface antigens, eliminating their protein coat, and modulating the host immune response, e.g., the maturation of the dendritic cells [15,16]. Malaria also manipulates the immune system of its mosquito hosts in two ways [17]. First, in the early stages of infection, it actively suppresses the encapsulation immune response within the mosquitoes. In addition, an indirect suppression occurs when mosquitoes (already infected or not) are fed with plasma of infected hosts. The underlying mechanisms are still unknown but there is a suggestion that this indirect suppression results from complex interactions between the vertebrate and the mosquito immune responses. The manipulation of the immune response is certainly an important way for the parasite to ensure its transmission.

### From phenotype to molecular mechanisms.

More generally, studying the molecular cross talks (e.g., with proteomics tools, see for instance [18]) between parasites and vectors at several stages of their interaction should not only permit us to understand the proximate mechanisms causing alterations in feeding behaviour, but also to potentially discover new ways in which parasites increase their transmission. Such an approach would appear promising to understand who is actually running the show: the parasite, the host, both, or neither. Similarly, this approach could bring relevant information when applied to interactions among pathogens and their vertebrate hosts [19]. To go further in this direction, an important hurdle that would also need to be overcome is the development of a population biology view, assessing the populational polymorphism in these processes. This implies an automation of molecular techniques in order to provide high throughput datasets.

### Conflict of interest.

Conflicts of interest in behavioural patterns naturally underlie any kind of manipulation. Mosquitoes, for example, would prefer to bite their hosts less frequently than what is optimal for transmission stages of the parasites [20]. Such conflicts can become quite complex if the parasite can manipulate several traits in its different hosts and change the manipulation according to its stage of development [21]. Conflicts between parasites are also expected when hosts harbour simultaneously transmissible and nontransmissible stages (both at the intraspecific and interspecific level). However, and unfortunately, very few studies consider these conflicts explicitly. How strong is the selection to manipulate the hosts? How strong is the selection to resist being manipulated? In addition to empirical approaches based on experimental infections, the understanding of these complex interspecific and intraspecific interactions would benefit from being explored from a theoretical point of view. Answers to such questions could bring studies of behavioural manipulation from interesting observations to predictive evolutionary biology.

Research on vector–pathogen interactions has *unfortunately* a bright future given the increasing preoccupations caused by the emergence and the reemergence of numerous infectious diseases. Because traditional medical approaches do not always provide suitable solutions (e.g., too expensive for the countries concerned), fundamental investigations of the ecology and the evolution of vector and pathogen interactions remain a key aspect of the research in human and veterinary health. Additionally, attention should be directed towards investigations in a field setting, as behaviour in the laboratory may not reflect precisely that which occurs naturally. Behavioural manipulations of vectors are phenomena so complex that one single method cannot totally describe or understand them. For this reason, future research should benefit from the expertise of different disciplines. Responses to the questions asked will indeed need the integration of the concepts and techniques from epidemiology, behavioural and evolutionary ecology, medicine, neurobiology, physiology, and molecular biology. Despite the difficulty of performing such pluridisciplinary approaches, these efforts will undoubtedly provide a much better basis for understanding the evolution of parasitic manipulation in vectors. Although speculative in appearance, each of the scenarios mentioned above is legitimate from ecological and evolutionary points of view. At least because of this, and also because the above hypotheses would considerably change the way we control and model the transmission of the most harmful pathogens affecting humans, they should be verified.

## References

[ppat-0020072-b001] Hurd H (2003). Manipulation of medically important insect vectors by their parasites. Annu Rev Entomol.

[ppat-0020072-b002] Lacroix R, Mukabana WR, Gouagna LC, Koella JC (2005). Malaria infection increases attractiveness of human to mosquitoes. PLoS Biol.

[ppat-0020072-b003] Molyneux DH, Jefferies D (1986). Feeding behaviour of pathogen-infected vectors. Parasitol.

[ppat-0020072-b004] Koella JC, Sørensen FL, Anderson R (1998). The malaria parasite Plasmodium falciparum increases the frequency of multiple feeding of its mosquito vector Anopheles gambiae. Proc R Soc Lond B.

[ppat-0020072-b005] Lehane M (2005). The biology of bloodsucking in insects. 2nd edition.

[ppat-0020072-b006] Liddell K (1976). Smell as a diagnostic marker. Postgrad Med J.

[ppat-0020072-b007] Brown R (1995). What is the role of the immune system in determining individually distinct body odours?. Int J Immunopharmacol.

[ppat-0020072-b008] Penn D, Potts WK (1998). Chemical signals and parasite-mediated sexual selection. Trends Ecol Evol.

[ppat-0020072-b009] Kavaliers M, Choleris E, Pfaff DW (2005). Genes, odours and the recognition of parasitized individuals by rodents. Trends Parasitol.

[ppat-0020072-b010] Baylis M, Mbwabi AL (1995). Feeding behaviour of tsetse flies *(Glossina pallidipes Austen)* on *Trypanosoma*-infected oxen in Kenya. Parasitol.

[ppat-0020072-b011] Moloo SK, Sabwa CL, Baylis M (2000). Feeding behaviour of Glossina pallidipes and *G. Morsitans centralis* on Boran cattle infected with Trypanosoma congolense or *T. Vivax* under laboratory conditions. Med Vet Entomol.

[ppat-0020072-b012] O'Shea B, Rebollar-Téllez E, Werd RD, Hamilton JGC, El Naiem D (2002). Enhanced sandfly attraction to Leishmania infected hosts. Trans R Soc Trop Med Hyg.

[ppat-0020072-b013] McLeod RG, von Reuß SH, Rahe JE, McIntosh R, Konig WA (2005). The pathogen causing Dutch elm disease makes host trees attract insect vectors. Proc R Soc Lond B.

[ppat-0020072-b014] Kelly DW (2001). Why are some people bitten more than others?. Trends Parasitol.

[ppat-0020072-b015] Zambrano-Villa S, Rosales-Borjas D, Carrero JC, Ortiz-Ortiz L (2002). How protozoan parasites evade the immune response. Trends Parasitol.

[ppat-0020072-b016] Urban BC, Ferguson DJ, Pain A, Willcox N, Plebanski M (1999). Plasmodium falciparum–infected erythrocytes modulate the maturation of dendritic cells. Nature.

[ppat-0020072-b017] Boëte C, Paul REL, Koella JC (2004). Direct and indirect immuno-suppression by a malaria parasite in its mosquito vector. Proc R Soc Lond B.

[ppat-0020072-b018] Biron DG, Marché L, Ponton F, Loxdale H, Galéotti N (2005). Behavioural manipulation in a grasshopper harbouring hairworm: A proteomics approach. Proc R Soc Lond B.

[ppat-0020072-b019] Stiles JK, Whittaker J, Sarfo BY, Thompson WE, Powell MD (2004). Trypanosome apoptotic factor mediates apoptosis in human brain vascular endothelial cells. Mol Biochem Parasitol.

[ppat-0020072-b020] Koella JC (1999). An evolutionary view of the interactions between anopheline mosquitoes and malaria parasites. Microbes Infect.

[ppat-0020072-b021] Anderson R, Koella JC, Hurd H (1999). The effect of *Plasmodium yoelii nigeriensis* infection on feeding persistence of Anopheles stephensi Liston throughout the sporogonic cycle. Proc R Soc of Lond B.

